# Human Lymphoid Stromal Cells Contribute to Polarization of Follicular T Cells Into IL-4 Secreting Cells

**DOI:** 10.3389/fimmu.2020.559866

**Published:** 2020-10-02

**Authors:** Jan Misiak, Rachel Jean, Stéphane Rodriguez, Laurent Deleurme, Thierry Lamy, Karin Tarte, Patricia Amé-Thomas

**Affiliations:** ^1^INSERM U1236, Univ Rennes, Etablissement Français du Sang Bretagne, LabEx IGO, Rennes, France; ^2^CHU de Rennes, Pôle Biologie, Rennes, France; ^3^Univ Rennes, CNRS, Inserm, BIOSIT (Biologie, Santé, Innovation Technologique de Rennes)—Unité Mixte de Service 3480, Rennes, France; ^4^CHU de Rennes, Service d’Hématologie Clinique, Rennes, France

**Keywords:** IL-4, fibroblastic reticular cells, follicular T cells, T follicular helper cells, follicular lymphoma

## Abstract

Fibroblastic reticular cells (FRCs) are the specialized lymphoid stromal cells initially identified as triggering T-cell recruitment and dynamic motion in secondary lymphoid organs. Interestingly, FRCs also display antigen presentation capacities and support lymphocyte survival. CXCR5^+^CD4^+^ follicular T cells are important players of B-cell maturation and antibody response. Our study reported that *in vitro*-differentiated FRC-like cells enhanced the growth of the whole CXCR5^+^CD4^+^ T-cell compartment, while enhancing IL-4 secretion specifically by the PD1^dim^CXCR5^+^CD4^+^ cell subset, in a Notch- and ICAM1/LFA1-dependent manner. In addition, we revealed that in follicular lymphoma (FL) tissues, previously identified as enriched for PD1^hi^CXCR5^hi^CD4^+^ mature follicular helper T cells, PD1^dim^CXCR5^+^CD4^+^ T cells displayed an enrichment for Notch and integrin gene signatures, and a Notch and ICAM-1-dependent overexpression of IL-4 compared to their non-malignant counterparts. These findings suggest that the crosstalk between FRCs and CXCR5^+^PD1^dim^CD4^+^ T cells may contribute to the FL IL-4 rich environment, thus providing new insights in FL lymphomagenesis.

## Introduction

T follicular helper cells (Tfh) have been described as a specialized mature CD4^pos^ T cell population involved in the development and maturation of germinal center (GC) B cells, enabling their survival and differentiation into high-affinity memory B cells and long-lived antibody-secreting cells (1, 2). Unlike other helper T cell compartments, follicular CD4^+^ T cells have been defined by their localization in secondary lymphoid organs (SLO) at the T/B border or within B-cell follicles, depending on their maturation stage. Human Tfh are defined by the expression of CXCR5, ICOS, PD1, and the GC transcription factor Bcl-6, which is essential for their development and maintenance (3, 4). It is now well known that these cells display a specific gene expression pattern supporting the hypothesis that they form a separate helper T-cell lineage, even if various subsets of Tfh producing different cytokines, such as IFN-γ, IL-4 or IL-17, have been described (5).

Besides B and T cells, lymphoid stromal cells represent important cell organizers enabling naive T- and B-cell recruitment in SLO. In particular, fibroblastic reticular cells (FRCs) are found in close contact with T cells allowing for their recruitment and dynamic motion along cytoplasmic extensions functioning as guiding paths (6). Several studies have featured the significant effect of FRCs on T cell survival (7) and their antigen-presenting cell properties (8), hypothesizing that they could play other roles in immunological responses by interacting more strongly with T cells than previously assumed. Accordingly, we decided to investigate the functional interactions between human FRCs and follicular CXCR5^+^CD4^+^ helper T cells.

Follicular lymphoma (FL), the most frequent indolent B-cell lymphoma, results from the malignant transformation of GC B cells. FL tumor cells remain strongly dependent on surrounding supportive cells (9, 10), including in particular Tfh (11–14) and lymphoid stromal cells (15, 16). Previous studies revealed that CXCR5^hi^PD1^hi^ GC-Tfh are abundant within FL lymph nodes (LN) (14) and support directly and indirectly FL B cell survival, especially through IL-4 overexpression (11, 17, 18). Moreover, immunohistochemistry studies of FL LNs revealed the presence of an overdeveloped and activated FRC network (19, 20). Thereby, we considered that FL could be a good model to fulfill the study of the interactions between FRCs and CXCR5^+^CD4^+^ follicular helper T cells.

In this study, we revealed a specific IL-4 over-secretion in a previously unexplored CXCR5^+^PD-1^dim^ CD4^+^ T-cell subset when cultured with FRCs, which involved ICAM1 and Notch pathways. This observation was of a particular interest to further the understanding of the FL supportive microenvironment and to highlight some of the mechanisms involved in the deregulated IL-4 expression.

## Materials and Methods

### Cell Samples

All tissues used for this study were obtained from subjects recruited under institutional review board approval and informed consent process according to the Declaration of Helsinki. Human stromal cells from SLO originated from pediatric patients undergoing routine tonsillectomy. Uncommitted tonsil stromal cells (TSCs) were obtained from Percoll-enriched cell fraction maintained in RPMI 1640-10% FCS, and were stimulated for 3 days by 20 ng/ml TNF-α and 100 ng/ml LT-α1β2 (RD Systems) to generate *in vitro*-differentiated FRC-like cells (FRCLs), as described previously (16). CXCR5^hi^PD-1^hi^ CD4^+^CD3^+^CD25^-^ and CXCR5^+^PD-1^dim^CD4^+^CD3^+^CD25^-^ were sorted (**Supplemental Figure 1**) and referred as GC-Tfh and R5-PD1^dim^ cells (4). It was assumed that few CD25^-^ Tfr cells were present in sorted samples. See **Supplemental Materials** and methods for details.

### Flow Cytometry Analysis

Monoclonal antibodies (mAbs) used are listed in **Supplemental Table S1**. Samples were acquired on a Gallios^®^ flow cytometer (Beckman Coulter) and singlets of viable cells were analyzed using Kaluza Analysis 1.3 software (Beckman Coulter), and ModFit LT (Verity Software House) for proliferation assessment.

### Microarray Data Analysis

Microarray hybridization (see **Supplemental Materials** and methods) and analysis were performed on purified R5-PD-1^dim^ and GC-Tfh isolated from 3 tonsils and 3 FL LN samples, and from 3 TSCs and 3 FRCLs. Differentially expressed genes were identified using a moderated t-test carried out with Chipster software (adjusted p-value <.05, log2 fold change >2). A pre-ranked Gene Set Enrichment Analysis (GSEA) was performed to evaluate the enrichment of Notch and integrin pathways in T-cell subsets. Moreover, GSEA preRanked was used to explore REACTOME, KEGG, PID, and BIOCARTA pathway databases. Pearson correlation matrix with pairwise complete observation was performed using R cor function on non-redundant transcript normalized values from TSCs, FRCLs, R5-PD-1^dim^, and GC-Tfh microarrays, and was visualized using R heatmap function. The top 20% most variable transcripts identified by statmod R package were retained to constitute the matrix. Microarray data are available under GEO accession number GSE157784 and GSE157801.

### Quantitative RT-PCR

Total RNA from R5-PD-1^dim^, GC-Tfh, and stromal cells was extracted using Nucleospin RNA kits (Macherey-Nagel) and either directly transcribed to cDNA with Superscript II reverse transcriptase (Invitrogen) or amplified with Ovation Pico SL (Nugen). For quantitative RT-PCR, assay-on-demand primers and probes (**Supplemental Table S2**) and the Taqman Universal Master Mix (Thermo Fisher Scientific) were used. Gene expression was measured using the StepOnePlus Real-Time PCR System or the ABI Prism 7900HT Sequence Detection System (Thermo Fisher Scientific). Appropriate housekeeping genes were selected as *B2M, CASC3*, and *18S* for T cells, and *CDKN1B* and *PUM1* for stromal cells.

### Proliferation and Survival Assays

For proliferation assays, sorted tonsil R5-PD-1^dim^ and GC-Tfh subsets were stained with CFSE and cultured in 10% FCS-RPMI 1640 alone or with pre-seeded TSCs or FRCLs (5:1 ratio) for 4 days with anti-CD3 (0.2 ug/ml) and anti-CD28 (0.2 ug/ml) stimulating antibodies (Sanquin). Cells were then trypsinized and stained with CD2 and CD105 to analyze CFSE^+^CD2^+^CD105^-^ T cells. For survival assays, sorted tonsil R5-PD-1^dim^ and GC-Tfh subsets were cultured in 10% FCS-RPMI 1640 alone or with preseeded TSCs or FRCLs (5:1 ratio) for 5 days, followed by CD2, CD105 and active caspase-3 staining according to the manufacturer’s instructions. Percentage of active caspase-3 negative cells was evaluated on CD2^+^CD105^-^ T cells.

### Cytokine Secretion Assay

Sorted tonsil or FL R5-PD-1^dim^ and GC-Tfh were cultured for 3 days in 10% FCS-RPMI 1640 with pre-seeded TSCs or FRCLs (5:1 ratio) in presence of anti-CD3 (0.2 ug/ml) and anti-CD28 (0.2 ug/ml) stimulating antibodies. After 3 days, a restimulation step was done with 100 ng/ml phorbol myristate acetate and 750 ng/ml ionomycin for 6 h, supplemented with GolgiPlug (Becton Dickinson) for the last 4 h. For inhibition experiments, Notch chemical inhibitor L685,458 (Sigma Aldrich) or blocking antibodies (bAbs) (**Supplemental Table 1**) were used. The percentage of singlet viable T cells producing IL-4, IL-21, and IFN-γ was determined by staining with live/dead fixable yellow dead cell stain (Thermo Fisher Scientific) and CD2, followed by fixation in paraformaldehyde 4% for 15min, permeabilization with saponin 0.5%, and staining for intracellular cytokines.

### Statistical Analysis

Statistical analyses were performed with Graphpad Prism 6 software suite (GraphPad Software) using non-parametric Wilcoxon test for matched pairs, or Mann Whitney U test.

## Results

### FRCs Stimulate the Expansion of Follicular CXCR5^+^ CD4^+^ T-Cell Compartments

Having identified two subsets of human CXCR5^+^CD4^+^ follicular T cells based on their differential expression of CXCR5 and PD-1 (**Supplemental Figure 1**), we decided to explore the impact of FRCs on both GC-Tfh and R5-PD1^dim^ cells. Indeed, FRCs express high levels of adhesion molecules, extracellular matrix components, and LN chemokines, and promote B and T cell recruitment, adhesion, and survival (7, 21, 22) in both T-cell zone, inter-follicular area, and at follicle border, the place of T-cell priming for Tfh differentiation. In addition, FRCLs obtained by *in vitro* differentiation of uncommitted TSCs have been proposed as a good model to perform functional FRC evaluation (16, 23).

**Figure 1:**
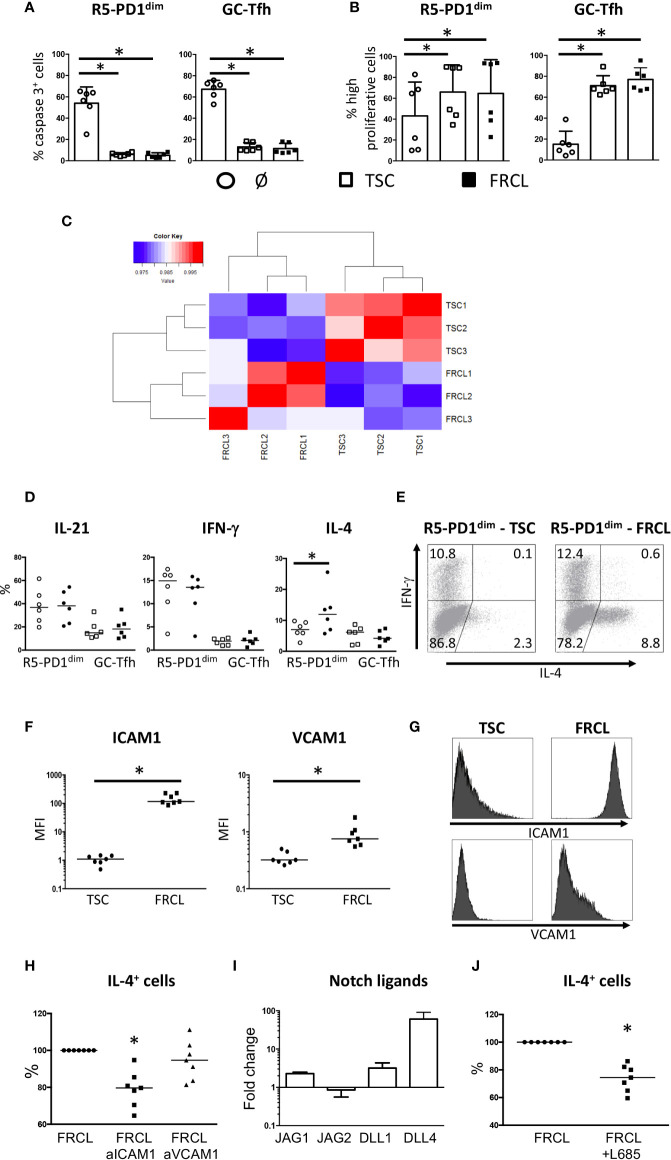
Survival, proliferation, and cytokine secretion of follicular CD4^+^ T cells cocultured with stromal cells. **(A, B)** Sorted tonsil R5-PD1^dim^ or GC-Tfh were cocultured alone (ø), or in presence of unpolarized TSCs, or FRCLs. Survival **(A)** and proliferation **(B)** were assessed by active caspase 3 and CFSE staining, respectively. Highly proliferative cells gathered cells that undergone more than one cycle of proliferation. *P < 0.05. **(C)** Pearson correlation matrix of microarray data obtained from 3 TSCs and 3 FRCLs. **(D)** IL-21, IFN-γ, and IL-4 secreting cells were assessed by flow cytometry. Results are expressed as the percentages of cytokine-secreting cells obtained from culture with TSCs (empty symbols) or FRCLs (full symbols). *P < 0.05. **(E)** Example of IFN-γ and IL-4 staining by flow cytometry for R5-PD1^dim^ cultured in the presence of TSCs or FRCLs. **(F)** Flow cytometry analysis of ICAM1 and VCAM1 expression at the cell surface of TSCs and FRCLs. MFI: mean fluorescence intensity. *P < 0.05. **(G)** One representative ICAM1 and VCAM1 flow cytometry staining for TSCs and FRCLs. **(H)** Sorted R5-PD1^dim^ cells were cocultured with FRCLs in the presence of anti-ICAM1 or anti-VCAM1 blocking antibodies before the quantification of IL-4 secreting cells by flow cytometry. Results are expressed as percentages of cytokine secretion for R5-PD1^dim^ cultured with FRCLs. Statistical analyses compared T-cell IL-4 secretion in FRCL+anti-ICAM1 and FRCL+anti-VCAM1 conditions versus that of FRCL alone as a control. *P < 0.05, n = 7. **(I)** *JAG1, JAG2, DLL1*, and *DLL4* expression in FRCLs compared with TSCs performed by quantitative RT-PCR. The arbitrary value of 1 has been assigned to TSCs (n = 3). **(J)** Sorted R5-PD1^dim^ were cocultured with FRCLs in presence or not of L685,458 before the quantification of IL-4 secreting cells by flow cytometry. Results are expressed as percentages of cytokine secretion for R5-PD1^dim^ cultured with FRCLs. *P < 0.05, n = 7.

Tonsil R5-PD1^dim^ and GC-Tfh were prone to die *ex vivo* when removed from their microenvironment and were efficiently rescued from death by coculture with both TSCs and FRCLs (**Figure 1A**). In addition, TSCs and FRCLs similarly enhanced the proliferation of R5-PD1^dim^ and GC-Tfh (**Figure 1B**). FRCLs and TSCs displayed thus similar capacities to sustain the growth of R5-PD1^dim^ and GC-Tfh. In order to decipher the specific impact of FRCLs on follicular CD4^+^ T cells, we then compared their gene expression profile (GEP) with those of TSCs. Unsupervised Pearson correlation performed on the top 20% most variable transcripts adequately segregated TSCs and FRCLs (**Figure 1C**). We then focused on genes overexpressed in FRCLs (**Supplemental Table 3**). Unexpectedly, pathway enrichment analysis using REACTOME database revealed a strong enrichment of FCRL signature for Notch-1 and Noctch-2 signaling. Moreover, several genes known to be involved in adhesion and antigen presentation to T cells were found in this FRCL signature and could impact CD4^+^ T-cell behavior. Among 733 genes, the adhesion molecule ICAM1 was the most upregulated gene. ICAM1 and CD58, which was also overexpressed in FRCL, are two molecules involved in adhesion process through binding of LFA-1 and CD2, respectively. Several inflammatory chemokines, such as CCL2, CCL5, CCL11, and CXCL10 were also found overexpressed, and could be involved in the recruitment of CD4^+^ activated T cells expressing CCR1, CCR2, CCR3, CCR4, CCR5, or CXCR3 (**Table 1**). In agreement to the previously demonstrated antigen-presenting cell properties of mouse LN stromal cells (8), we also observed an overexpression of CD74, which is involved in the formation and transport of MHC class II protein (24), as well as CD83 which is known to deliver costimulatory signals for naive and memory T-cell activation (25). We also revealed a high expression of immunosuppressive molecules such as HLA-G and CD274, in agreement with the recently proposed role of FRCs in immune tolerance (26–28). Finally, we found an overexpression of cytokines involved in CD4^+^ T-cell development: IL-15 involved in CD4^+^ T-cell homeostasis (29), IL-6 involved in Tfh differentiation (30), and IL-33 leading to secretion of Th2 associated cytokines (IL-4, IL-5, IL-13) and increase of immunoglobulin levels (31, 32). Overall, our microarray data suggest that human FRCs can modulate follicular CD4^+^ T-cell behavior.

**Table 1:** Selected genes upregulated in FRC-like cells (FRCLs) compared to uncommitted tonsil stromal cells (TSCs).

Probeset ID	Gene Symbol	Fold Change FRCL/TSC	p-value
209619_at	CD74	5,5	0,0033
204440_at	CD83	13,7	0,0195
202638_s_at	ICAM1	1018,8	0,0004
217371_s_at	IL15	3,5	0,0060
209821_at	IL33	5	0,0185
205207_at	IL6	61,6	0,0021

### FRCs Specifically Enhance IL-4 Secretion by R5-PD1^dim^ T Cells in a Notch- and ICAM1-Dependent Manner

It is now well-described that different subsets of follicular T cells could be defined based on their cytokine profile expression (33, 34). Comparing R5-PD1^dim^ cultured with TSCs *versus* FRCLs, no modification of IL-21 and IFN-γ secretion was observed (**Figure 1D**). Similarly, a coculture of GC-Tfh with either TSCs or FRCLs did not affect their secretion of IL-21 and IFN-γ, despite a lower overall cytokine secretion ability compared to R5-PD1^dim^ (**Figure 1D**). Interestingly, we found that in presence of FRCLs, R5-PD1^dim^ specifically upregulated IL-4 production, unlike paired GC-Tfh (**Figures 1D, E**). Together with the similar impact of TSCs and FCRLs on R5-PD1^dim^ proliferation and survival (**Figure 1A**), this finding suggests specific signaling pathways between FRCLs and R5-PD1^dim^, involving molecules favoring IL-4 production.

It has been recently described in mice that the ICAM-1-binding CD11a/CD18 heterodimer (LFA-1) controls Tfh generation and maintenance, and is involved in the development of IL-4 producing Tfh and Th2 cells during helminth infection (35). Of note, *ICAM1* was the most upregulated gene in FRCLs compared to TSCs, and was highly expressed, together with VCAM1, on FRCL membrane (**Figures 1F, G**). In addition, as already described in the case of other CD4^+^ T cell subsets (36), tonsil R5-PD1^dim^ expressed CD49d, CD29, CD11a, and CD18 integrin subunits (data not shown). Interestingly, we reported a significant decrease of IL-4 secretion by R5-PD1^dim^ cocultured with FRCLs in the presence of anti-ICAM1, and not anti-VCAM1, blocking Abs (**Figure 1H**, **Supplemental Figure 2**). These findings highlight a specific involvement of ICAM1/LFA-1 pathway in IL-4 overexpression of R5-PD1^dim^ in contact with FRCLs.

Furthermore, it has been demonstrated that Notch affects Tfh differentiation (37, 38), and mounting evidence suggest that Notch signaling is involved in activation of IL-4 secretion by Tfh. Indeed, activation of IL-4 expression in mouse Tfh was shown to be predominantly dependent on the 3’ enhancer CNS2 (39), and Notch intracellular domain was previously shown to bind selectively to CNS2 (40). Interestingly, *JAG1*, *DLL1*, and *DLL4* Notch ligands were overexpressed in FRCLs compared to TSCs (**Figure 1I**). Using L685,458, a gamma-secretase inhibitor that fully blocks Notch signaling, a significant decrease of IL-4 secretion was observed for R5-PD1^dim^ cocultured with FRCLs (**Figure 1J**, **Supplemental Figure 2**). This result suggests that in addition to ICAM1, Notch signaling also participates in IL-4 secretion of R5-PD1^dim^ cells in contact with FRCLs.

### FL LN-Infiltrating R5-PD1^dim^ Cells Secrete High Levels of IL-4

**Figure 2:**
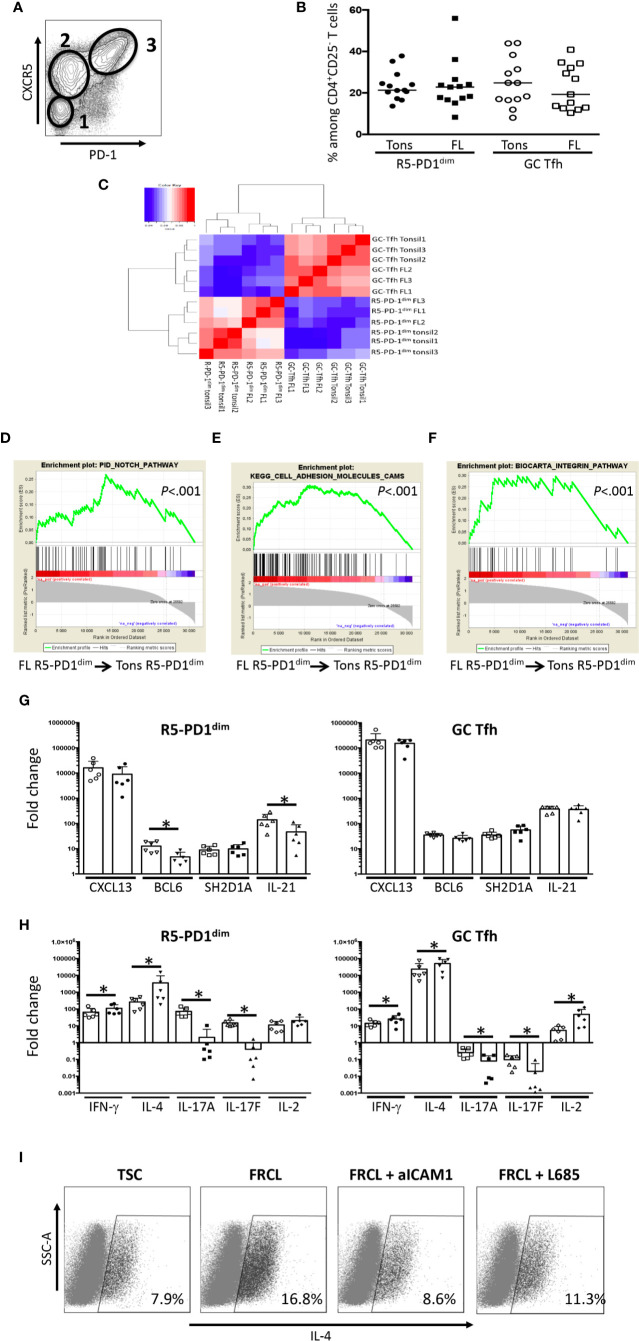
Characterization of R5-PD1^dim^ infiltrating FL LN as an IL-4-secreting subset. **(A)** PD1 and CXCR5 expression of viable FL LN CD4^+^CD25^-^ T cells as evaluated by flow cytometry. Shown is one example of staining. 1: non Tfh; 2: R5-PD1^dim^; 3: GC-Tfh. **(B)** Frequencies of R5-PD1^dim^ and GC-Tfh among viable CD4^+^CD25^-^ T cells in tonsils (Tons) and FL LN (FL). **(C)** Pearson correlation matrix of microarray data obtained from GC-Tfh and R5-PD1^dim^ subsets isolated from tonsils and FL LN. **(D–F)** Gene expression profile of R5-PD1^dim^ isolated from FL LN (FL) and tonsils (Tons) were compared, and GSEA enrichment plots for PID Notch pathway **(D)**, Kegg cell adhesion molecules **(E)**, and Biocarta integrin pathway **(F)** were drawn. The green curve represents the running sum of the weighted enrichment score. **(G, H)** Quantitative RT-PCR analyses were made on sorted R5-PD1^dim^ and GC-Tfh isolated from tonsil (Tons) and FL LN (FL). Tonsil and FL LN samples are represented by white and black symbols, respectively. The arbitrary value of 1 has been assigned to blood naive CD4^+^ T cells, used as an internal control. *P < 0.05. **(I)** Sorted FL R5-PD1^dim^ were cocultured with TSCs or FRCLs in the presence or not of ICAM1 blocking antibodies or L685,458 before the quantification of IL-4 secreting cells by flow cytometry. Shown is one experiment out of two.

Based on FL LN histochemistry studies revealing an overdeveloped FRC network (19), an over-representation of GC-Tfh (11, 14), and an IL-4 rich microenvironment (18), we hypothesized that stromal cell/follicular CD4^+^ T-cell interactions could play a key role in FL lymphomagenesis. Like in tonsils, FL CD4^+^ T-cell characterization revealed the presence of three CD4^+^ T-cell subsets based on CXCR5 and PD-1 expression: a CXCR5^low^PD-1^low^ subset representing FL non-Tfh, and two CXCR5 expressing subsets, CXCR5^+^PD-1^dim^ (FL R5-PD1^dim^) and CXCR5^+/hi^PD-1^hi^ (FL GC-Tfh) (**Figure 2A**). Of note, tonsils and FL LN samples displayed similar R5-PD1^dim^ and GC-Tfh frequencies (**Figure 2B**).

In order to highlight the specific features of FL follicular T cells, we analyzed the GEP of R5-PD1^dim^ and GC-Tfh subsets isolated from FL LN and tonsils. Strikingly, an unsupervised Pearson correlation performed on the top 20% most variable genes segregated adequately follicular CD4^+^ T not only based on their phenotype, *i.e.* R5-PD1^dim^ versus GC-Tfh, but also based on their malignant versus non-malignant origin (**Figure 2C**). However, supervised stringent analysis identified only few differentially expressed genes between tonsil and FL LN R5-PD1^dim^: 47 upregulated and 38 downregulated genes (**Supplemental Table S4**). Using Gene Set Enrichment Analysis (GSEA) and looking for *a priori* defined genelists, three genesets were found enriched in FL R5-PD1^dim^: PID Notch pathway, KEGG cell adhesion molecules and Biocarta Integrin Pathway (**Figures 2D–F**); the latter two comprising all described adhesion molecules and integrin downstream molecules, respectively. To have a more accurate phenotype of FL LN CD4^+^ T-cell subsets, expression of typical Tfh genes was evaluated by quantitative RT-PCR. Even though FL R5-PD1^dim^ expressed lower levels of *BCL6* and *IL21* compared to tonsil R5-PD1^dim^, the expression of those two genes was lower than in paired FL GC-Tfh (**Figure 2G**), and higher than in paired FL non-Tfh (data not shown). This finding allowed us to hypothesize that R5-PD1^dim^ could correspond to Tfh precursors. Like FL GC-Tfh, FL R5-PD1^dim^ expressed *LTA* and *LTB*, as well as high levels of *TNFA* (**Supplemental Figure 3**), all known to be involved in FRC differentiation (41), suggesting that these FL R5-PD1^dim^ could participate, as well as FL GC-Tfh (16) to the FRC network expansion found in FL LNs. Finally, we highlighted an *IFN-γ*^hi^ *IL-4*^hi^ *IL-17*^low^ phenotype in FL R5-PD1^dim^ besides the already described specific *IFN-γ*^hi^ *IL-4*^hi^ *IL-2*^hi^ *IL-17*^low^ profile of FL GC-Tfh (**Figure 2H**) (11, 13). Interestingly, *IL-4* was the most differentially overexpressed cytokine in FL R5-PD1^dim^ compared to tonsil counterpart. We hypothesized that this IL-4 overexpression could be linked to the stimulation of FL-Tfh with integrins and/or Notch ligands expressed by the expanded FL-FRC network. In agreement, a significant decrease of IL-4 secretion was observed in purified FL R5-PD1^dim^ cultured with FRCLs in the presence of either ICAM1 blocking antibodies or L685,458 Notch inhibitor (**Figure 2I**).

## Discussion

The primary goal of this study was to define the interactions between lymphoid stromal cells, and follicular CD4^+^ T cells. Numerous studies revealed an immunosuppressive effect of stromal cells on CD4^+^ helper T cells [reviewed in Duffy MM et al. (42)]. In particular, we and others have shown that IFN-γ secreted by CD4^+^ T cells induce the expression of the tryptophane-catabolizing enzyme indoleamine 2,3-dioxygenase (IDO) in human stromal cells, inhibiting CD8^+^ and CD4^+^ T-cell proliferation (43, 44). Although a subset of both GC-Tfh and R5-PD1^dim^ cells secreted IFN-γ, a higher proliferation rate has been observed in this study during Tfh and stromal cell cocultures suggesting that follicular CD4^+^ T cells exhibit a specific mechanism of protection against IDO activity.

Moreover, this study highlights a higher frequency of IL-4^+^ R5-PD1^dim^ in the presence of FRCLs compared to unpolarized TSCs, whereas the proportions of IFN-γ^+^ and IL-21^+^ R5-PD1^dim^ were unaffected. Besides GC-Tfh, well-known to produce IL-4, an important cytokine involved in T-dependent antibody responses (45), R5-PD1^dim^ may thus also participate in the IL-4 rich environment in SLO. Since no difference in survival and proliferation rates was observed between R5-PD1^dim^ cocultured with TSCs and FRCL, the activation of signaling pathways enhancing IL-4 secretion by R5-PD1^dim^ was more likely than a specific anti-apoptotic/pro-proliferative effect on IL-4^+^ R5-PD1^dim^. Both Notch and ICAM1/LFA1 were involved in this IL-4 over-secretion. Interestingly, expression of Notch ligands by FL-infiltrating stromal cells has already been involved in their capacity to polarize recruited monocytes into supportive tumor-associated macrophages (46). We can speculate that Batf or c-maf (47), that have been described as important for IL-4 synthesis in follicular CD4^+^ T cells, could be engaged in stimulated follicular T cells, even if no correlation between their gene expression and IL-4 gene expression in R5-PD1^dim^ has been found (data not shown).

An important hallmark of FL is the major role of the microenvironment in malignant B cell survival, growth, and drug resistance (10). Deciphering the specific features of FL cell niche components and understanding their relationship with neoplastic cells should be helpful in improving the design of targeted therapies in this still fatal malignancy. Helper T cells represent a prominent non-malignant cell subset in FL. GEP of total CD4^pos^ T cells has revealed an altered expression of numerous genes related to T-cell activation, motility, and polarization, including an upregulation of *IL4* compared to tonsil T helper cells (48). However, such approaches do not consider the functional diversity of CD4^+^ T-cell subsets. FL GC-Tfh have been described as a major component of the FL niche due to their localization in malignant follicles and their malignant B cell supportive properties, in part due to their high IL-4 secretion (16). Here, we further reveal that R5-PD1^dim^ could also be an important actor in the formation of the peculiar FL LN microenvironment. Indeed, FL R5-PD1^dim^ displayed an IL-4 over-secretion, probably caused in part by their interaction with FL-FRCs, in a Notch- and ICAM-1-dependent manner. More extensive experiments need to be performed to decipher if IL-4-secreting FL GC-Tfh actually derive from FL R5-PD1^dim^. Furthermore, FL LNs display an overdeveloped FRC network, that have been demonstrated to be supportive for malignant FL B cells (19), and could be in part a consequence of TNF-α secretion by FL R5-PD1^dim^ and FL GC-Tfh. Overall, follicular CD4^+^ T cells seems to be central supportive cells for malignant FL B cells.

In summary, we report a close collaboration between two poorly explored compartments of SLO, FRCs and R5-PD1^dim^, for IL-4 secretion in both physiological conditions and in the context of FL. This study enhances our current understanding of the multipartite cell interactions occurring in the FL cell niche and encourages their deeper characterization in order to develop targeted therapeutic agents.

## Data Availability Statement

The datasets presented in this study can be found in online repositories. The names of the repository/repositories and accession number(s) can be found in the article/**Supplementary Material**.

## Ethics Statement

The studies involving human participants were reviewed and approved by French Minister authorization DC-2016-2565. The patients/participants provided their written informed consent to participate in this study.

## Author Contributions

JM designed and performed experiments, analyzed and interpreted data and wrote the paper. RJ and LD performed experiments, analyzed and interpreted data. SR designed and performed experiments, analyzed and interpreted data and reviewed the paper. TL interpreted data and reviewed the paper. KT designed the research, analyzed and interpreted data and wrote the paper. PA-T designed the research, designed experiments, analyzed and interpreted data and wrote the paper. All authors contributed to the article and approved the submitted version.

## Funding

This work was supported by research grants from the Institut National du Cancer (INCA_6530), and the Fondation ARC pour la Recherche sur le Cancer (Grant PGA1 RF20170205386). JM has been funded by of a doctoral fellowship from the FP7 Marie Curie Initial Training Network (ITN 289720 Stroma).

## Conflict of Interest

The authors declare that the research was conducted in the absence of any commercial or financial relationships that could be construed as a potential conflict of interest.

## References

[1] Bryant VL Ma CS Avery DT Li Y Good KL Corcoran LM Cytokine-mediated regulation of human B cell differentiation into Ig-secreting cells: predominant role of IL-21 produced by CXCR5+ T follicular helper cells. J Immunol (2007) 179:8180–90. 10.4049/jimmunol.179.12.8180 18056361

[2] Crotty S A brief history of T cell help to B cells. Nat Rev Immunol (2015) 15:185–9. 10.1038/nri3803 PMC4414089 25677493

[3] Rasheed A-U Rahn H-P Sallusto F Lipp M Müller G Follicular B helper T cell activity is confined to CXCR5(hi)ICOS(hi) CD4 T cells and is independent of CD57 expression. Eur J Immunol (2006) 36:1892–903. 10.1002/eji.200636136 16791882

[4] Misiak J Tarte K Amé-Thomas P Flow cytometric detection and isolation of human tonsil or lymph node T follicular helper cells. Methods Mol Biol (2015) 1291:163–73. 10.1007/978-1-4939-2498-1_14 25836310

[5] Cannons JL Lu KT Schwartzberg PL T follicular helper cell diversity and plasticity. Trends Immunol (2013) 34:200–7. 10.1016/j.it.2013.01.001 PMC3646926 23395212

[6] Denton AE Linterman MA Stromal networking: cellular connections in the germinal centre. Curr Opin Immunol (2017) 45:103–11. 10.1016/j.coi.2017.03.001 28319729

[7] Link A Vogt TK Favre S Britschgi MR Acha-Orbea H Hinz B Fibroblastic reticular cells in lymph nodes regulate the homeostasis of naive T cells. Nat Immunol (2007) 8:1255–65. 10.1038/ni1513 17893676

[8] Turley SJ Fletcher AL Elpek KG The stromal and haematopoietic antigen-presenting cells that reside in secondary lymphoid organs. Nat Rev Immunol (2010) 10:813–25. 10.1038/nri2886 21088682

[9] Verdière L Mourcin F Tarte K Microenvironment signaling driving lymphomagenesis. Curr Opin Hematol (2018) 25:335–45. 10.1097/MOH.0000000000000440 29746265

[10] Amé-Thomas P Tarte K The yin and the yang of follicular lymphoma cell niches: role of microenvironment heterogeneity and plasticity. Semin Cancer Biol (2014) 24:23–32. 10.1016/j.semcancer.2013.08.001 23978491

[11] Ame-Thomas P Le Priol J Yssel H Caron G Pangault C Jean R Characterization of intratumoral follicular helper T cells in follicular lymphoma: role in the survival of malignant B cells. Leukemia (2012) 26:1053–63. 10.1038/leu.2011.301 PMC3428269 22015774

[12] Epron G Ame-Thomas P Le Priol J Pangault C Dulong J Lamy T Monocytes and T cells cooperate to favor normal and follicular lymphoma B-cell growth: role of IL-15 and CD40L signaling. Leukemia (2012) 26:139–48. 10.1038/leu.2011.179 21788945

[13] Amé-Thomas P Hoeller S Artchounin C Misiak J Braza MS Jean R CD10 delineates a subset of human IL-4 producing follicular helper T cells involved in the survival of follicular lymphoma B cells. Blood (2015) 125:2381–5. 10.1182/blood-2015-02-625152 PMC4401349 25733581

[14] Townsend W Pasikowska M Yallop D Phillips EH Patten PEM Salisbury JR The architecture of neoplastic follicles in follicular lymphoma; analysis of the relationship between the tumor and follicular helper T-cells. Haematologica (2020) 105:1593–603. 10.3324/haematol.2019.220160 PMC7271595 31537685

[15] Maby-El Hajjami H Amé-Thomas P Pangault C Tribut O DeVos J Jean R Functional alteration of the lymphoma stromal cell niche by the cytokine context: role of indoleamine-2,3 dioxygenase. Cancer Res (2009) 69:3228–37. 10.1158/0008-5472.CAN-08-3000 19276371

[16] Amé-Thomas P Maby-El Hajjami H Monvoisin C Jean R Monnier D Caulet-Maugendre S Human mesenchymal stem cells isolated from bone marrow and lymphoid organs support tumor B-cell growth: role of stromal cells in follicular lymphoma pathogenesis. Blood (2007) 109:693–702. 10.1182/blood-2006-05-020800 16985173

[17] Amin R Mourcin F Uhel F Pangault C Ruminy P Dupré L DC-SIGN-expressing macrophages trigger activation of mannosylated IgM B-cell receptor in follicular lymphoma. Blood (2015) 126:1911–20. 10.1182/blood-2015-04-640912 PMC4626662 26272216

[18] Pangault C Ame-Thomas P Ruminy P Rossille D Caron G Baia M Follicular lymphoma cell niche: identification of a preeminent IL-4-dependent T(FH)-B cell axis. Leukemia (2010) 24:2080–9. 10.1038/leu.2010.223 PMC3317889 20944673

[19] Thomazy VA Vega F Medeiros LJ Davies PJ Jones D Phenotypic modulation of the stromal reticular network in normal and neoplastic lymph nodes: tissue transglutaminase reveals coordinate regulation of multiple cell types. Am J Pathol (2003) 163:165–74. 10.1016/S0002-9440(10)63640-1 PMC1868169 12819021

[20] Pandey S Mourcin F Marchand T Nayar S Guirriec M Pangault C IL-4/CXCL12 loop is a key regulator of lymphoid stroma function in follicular lymphoma. Blood (2017) 129:2507–18. 10.1182/blood-2016-08-737239 28202459

[21] Cremasco V Woodruff MC Onder L Cupovic J Nieves-Bonilla JM Schildberg FA B cell homeostasis and follicle confines are governed by fibroblastic reticular cells. Nat Immunol (2014) 15:973–81. 10.1038/ni.2965 PMC4205585 25151489

[22] Krishnamurty AT Turley SJ Lymph node stromal cells: cartographers of the immune system. Nat Immunol (2020) 21:369–80. 10.1038/s41590-020-0635-3 32205888

[23] Katakai T Hara T Sugai M Gonda H Shimizu A Lymph node fibroblastic reticular cells construct the stromal reticulum via contact with lymphocytes. J Exp Med (2004) 200:783–95. 10.1084/jem.20040254 PMC2211971 15381731

[24] Wilson KM Labeta MO Pawelec G Fernandez N Cell-surface expression of human histocompatibility leucocyte antigen (HLA) class II-associated invariant chain (CD74) does not always correlate with cell-surface expression of HLA class II molecules. Immunology (1993) 79:331–5. PMC1421856 8344710

[25] Tze LE Horikawa K Domaschenz H Howard DR Roots CM Rigby RJ CD83 increases MHC II and CD86 on dendritic cells by opposing IL-10-driven MARCH1-mediated ubiquitination and degradation. J Exp Med (2011) 208:149–65. 10.1084/jem.20092203 PMC3023131 21220452

[26] Hirosue S Dubrot J Modes of Antigen Presentation by Lymph Node Stromal Cells and Their Immunological Implications. Front Immunol (2015) 6:446:446. 10.3389/fimmu.2015.00446 26441957 PMC4561840

[27] Baptista AP Roozendaal R Reijmers RM Koning JJ Unger WW Greuter M Lymph node stromal cells constrain immunity via MHC class II self-antigen presentation. Elife (2014) 3:e24226. 10.7554/eLife.04433 PMC4270074 25407678

[28] Abe J Shichino S Ueha S Hashimoto S-I Tomura M Inagaki Y Lymph node stromal cells negatively regulate antigen-specific CD4+ T cell responses. J Immunol (2014) 193:1636–44. 10.4049/jimmunol.1302946 25024385

[29] Chen X-L Bobbala D Cepero Donates Y Mayhue M Ilangumaran S Ramanathan S IL-15 trans-presentation regulates homeostasis of CD4(+) T lymphocytes. Cell Mol Immunol (2014) 11:387–97. 10.1038/cmi.2014.13 PMC4085515 24658435

[30] Choi YS Eto D Yang JA Lao C Crotty S Cutting edge: STAT1 is required for IL-6-mediated Bcl6 induction for early follicular helper cell differentiation. J Immunol (2013) 190:3049–53. 10.4049/jimmunol.1203032 PMC3626564 23447690

[31] Schmitz J Owyang A Oldham E Song Y Murphy E McClanahan TK IL-33, an interleukin-1-like cytokine that signals via the IL-1 receptor-related protein ST2 and induces T helper type 2-associated cytokines. Immunity (2005) 23:479–90. 10.1016/j.immuni.2005.09.015 16286016

[32] Peine M Marek RM Löhning M IL-33 in T Cell Differentiation, Function, and Immune Homeostasis. Trends Immunol (2016) 37:321–33. 10.1016/j.it.2016.03.007 27055914

[33] Crotty S Follicular helper CD4 T cells (TFH). Annu Rev Immunol (2011) 29:621–63. 10.1146/annurev-immunol-031210-101400 21314428

[34] Weinstein JS Herman EI Lainez B Licona-Limón P Esplugues E Flavell R TFH cells progressively differentiate to regulate the germinal center response. Nat Immunol (2016) 17:1197–205. 10.1038/ni.3554 PMC5030190 27573866

[35] Meli AP Fontés G Avery DT Leddon SA Tam M Elliot M The Integrin LFA-1 Controls T Follicular Helper Cell Generation and Maintenance. Immunity (2016) 45:831–46. 10.1016/j.immuni.2016.09.018 PMC5672956 27760339

[36] Damle NK Klussman K Leytze G Aruffo A Linsley PS Ledbetter JA Costimulation with integrin ligands intercellular adhesion molecule-1 or vascular cell adhesion molecule-1 augments activation-induced death of antigen-specific CD4+ T lymphocytes. J Immunol (1993) 151:2368–79. 7689606

[37] Fasnacht N Huang H-Y Koch U Favre S Auderset F Chai Q Specific fibroblastic niches in secondary lymphoid organs orchestrate distinct Notch-regulated immune responses. J Exp Med (2014) 211:2265–79. 10.1084/jem.20132528 PMC4203954 25311507

[38] Auderset F Schuster S Fasnacht N Coutaz M Charmoy M Koch U Notch signaling regulates follicular helper T cell differentiation. J Immunol (2013) 191:2344–50. 10.4049/jimmunol.1300643 23918982

[39] Harada Y Tanaka S Motomura Y Harada Y Ohno S-I Ohno S The 3’ enhancer CNS2 is a critical regulator of interleukin-4-mediated humoral immunity in follicular helper T cells. Immunity (2012) 36:188–200. 10.1016/j.immuni.2012.02.002 22365664

[40] Tanaka S Tsukada J Suzuki W Hayashi K Tanigaki K Tsuji M The interleukin-4 enhancer CNS-2 is regulated by Notch signals and controls initial expression in NKT cells and memory-type CD4 T cells. Immunity (2006) 24:689–701. 10.1016/j.immuni.2006.04.009 16782026

[41] Mebius RE Organogenesis of lymphoid tissues. Nat Rev Immunol (2003) 3:292–303. 10.1038/nri1054 12669020

[42] Duffy MM Ritter T Ceredig R Griffin MD Mesenchymal stem cell effects on T-cell effector pathways. Stem Cell Res Ther (2011) 2:34. 10.1186/scrt75 21861858 PMC3219065

[43] Ménard C Pacelli L Bassi G Dulong J Bifari F Bezier I Clinical-grade mesenchymal stromal cells produced under various good manufacturing practice processes differ in their immunomodulatory properties: standardization of immune quality controls. Stem Cells Dev (2013) 22:1789–801. 10.1089/scd.2012.0594 PMC3668498 23339531

[44] Meisel R Zibert A Laryea M Göbel U Däubener W Dilloo D Human bone marrow stromal cells inhibit allogeneic T-cell responses by indoleamine 2,3-dioxygenase-mediated tryptophan degradation. Blood (2004) 103:4619–21. 10.1182/blood-2003-11-3909 15001472

[45] McGuire HM Vogelzang A Warren J Loetsch C Natividad KD Chan TD IL-21 and IL-4 Collaborate To Shape T-Dependent Antibody Responses. J Immunol (2015) 195:5123–35. 10.4049/jimmunol.1501463 26491200

[46] Guilloton F Caron G Ménard C Pangault C Amé-Thomas P Dulong J Mesenchymal stromal cells orchestrate follicular lymphoma cell niche through the CCL2-dependent recruitment and polarization of monocytes. Blood (2012) 119:2556–67. 10.1182/blood-2011-08-370908 22289889

[47] Sahoo A Alekseev A Tanaka K Obertas L Lerman B Haymaker C Batf is important for IL-4 expression in T follicular helper cells. Nat Commun (2015) 6:7997. 10.1038/ncomms8997 26278622 PMC4557271

[48] Kiaii S Clear AJ Ramsay AG Davies D Sangaralingam A Lee A Follicular lymphoma cells induce changes in T-cell gene expression and function: potential impact on survival and risk of transformation. J Clin Oncol (2013) 31:2654–61. 10.1200/JCO.2012.44.2137 PMC3709054 23775959

