# Supplementary figures and images for: Human Lymphoid Stromal Cells Contribute to Polarization of Follicular T Cells Into IL-4 Secreting Cells
Source: Front Immunol. 2020 Oct 2;11:559866. doi: 10.3389/fimmu.2020.559866 (PMC7562812; doi:10.3389/fimmu.2020.559866)

# Supplemental Figure 1

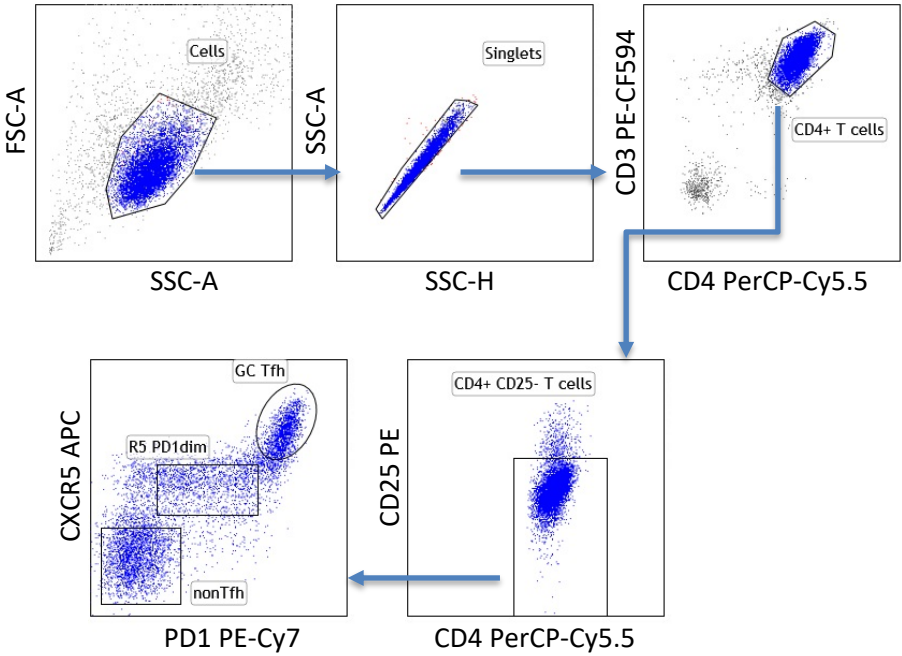

Supplement: Supplementary file 1 [file DataSheet_1.pdf]

Supplemental Figure 2

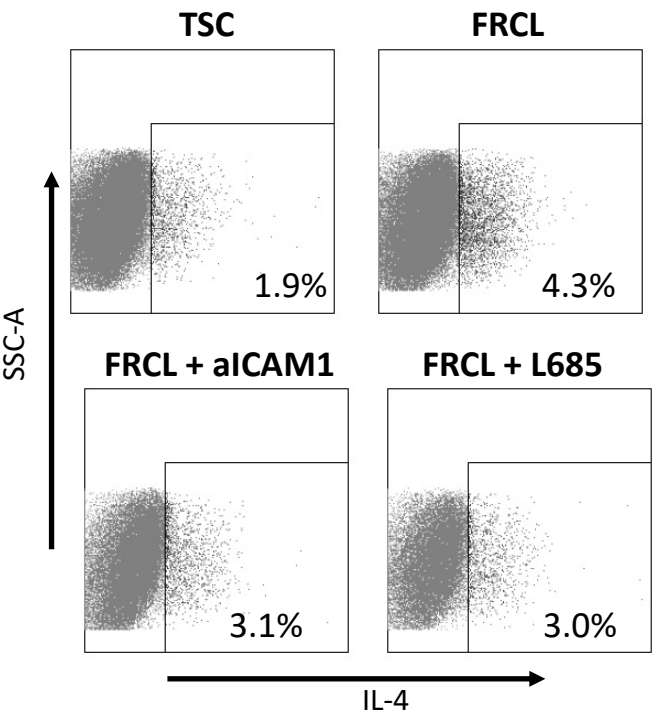

Supplement: Supplementary file 2 [file DataSheet_2.pdf]

Supplemental Figure 3

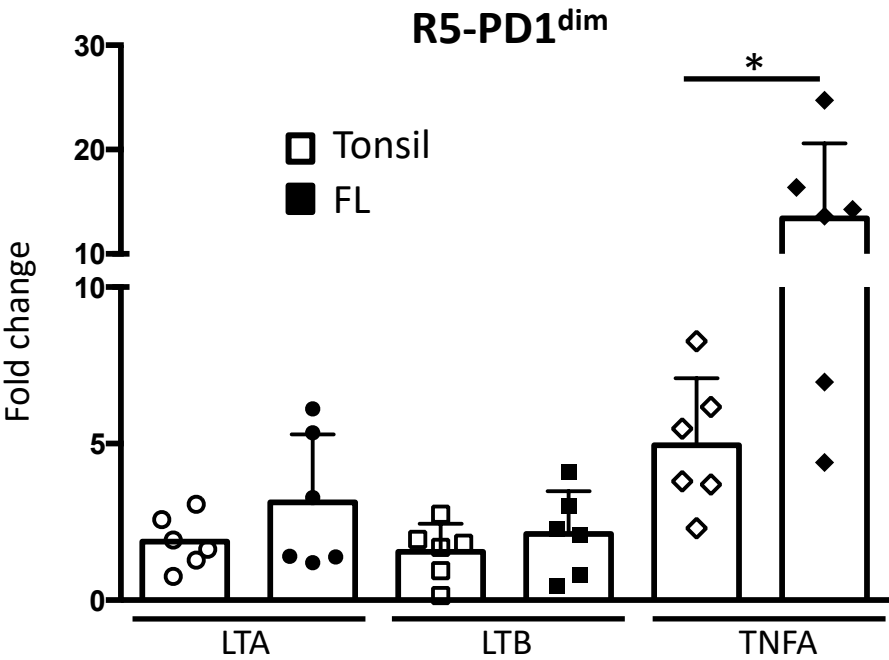

Supplement: Supplementary file 3 [file DataSheet_3.pdf]
