# Supplementary material for: Human Lymphoid Stromal Cells Contribute to Polarization of Follicular T Cells Into IL-4 Secreting Cells
Source: Front Immunol. 2020 Oct 2;11:559866. doi: 10.3389/fimmu.2020.559866 (PMC7562812; doi:10.3389/fimmu.2020.559866)
Supplement: Supplementary file 4 [file DataSheet_4.doc]

**SUPPLEMENTAL FIGURE LEGENDS**

**Supplemental figure 1: Gating strategy for follicular CD4 T cell subset sorting**

Enriched CD4^+^ T cells were stained with anti-human CD3, CD4, CD25, CXCR5 and PD1. GC Tfh and R5-PD1dim cells were sorted from CD3^+^CD4^+^CD25^-^ T cells based on their respective CXCR5 and PD1 expression levels.

**Supplemental figure 2: IL-4 secretion of R5-PD1^dim^ in presence of FRCLs and ICAM1 or Notch inhibitors**

Sorted R5-PD1^dim^ were cocultured with TSCs or FRCLs in presence or not of ICAM1 blocking antibodies or L685,458 before the quantification of IL-4 secreting cells by flow cytometry. One representative experiment.

**Supplemental figure 3: Expression of *TNFA*, *LTA* and *LTB* by FL follicular CD4^+^ T cells**

*LTA*, *LTB* and *TNFA* gene expression evaluated by Q-PCR of R5-PD1^dim^ cells isolated from tonsil and FL LN samples. The arbitrary value of 1 has been assigned to CXCR5^-^PD1^-^ non-Tfh cells. * P<0.05.
