# Supplementary material for: Human Lymphoid Stromal Cells Contribute to Polarization of Follicular T Cells Into IL-4 Secreting Cells
Source: Front Immunol. 2020 Oct 2;11:559866. doi: 10.3389/fimmu.2020.559866 (PMC7562812; doi:10.3389/fimmu.2020.559866)
Supplement: Supplementary file 5 [file DataSheet_5.doc]

**SUPPLEMENTAL MATERIAL AND METHODS**

**Cell samples**

Human stromal cells from secondary lymphoid organs were obtained from pediatric patients undergoing routine tonsillectomy, as previously described(1). Briefly, tonsils were cut into pieces and dissociated mechanically and enzymatically (collagenase IV and DNAse) before enrichment of stromal cells through discontinuous Percoll gradient. Cells were subsequently cultured in 10% FCS-RPMI 1640 (Thermo Fisher Scientific) with penicillin/streptomycin and are called uncommitted tonsil stromal cells (TSCs). FRC-like cells (FRCLs) were obtained from TSCs stimulated 3 days with TNF-α (20ng/mL) and lymphotoxin-α1β2 (LT-α1β2, 100 ng/mL, R&D Systems). Human CD4^+^ T cells were obtained from FL LN biopsies and non-malignant tonsils by perfusion followed by enrichment for mononuclear cells using Ficoll gradient and MACS sorting of CD19/CD14/CD8/CD16 negative cells. Mature GC-Tfh (CD3^+^CD4^+^CXCR5^+/hi^PD1^hi^CD25^-^), R5-PD1^dim^ (CD3^+^CD4^+^CXCR5^+^PD1^dim^CD25^-^) cells were then sorted with a FACSAria flow cytometer (Becton Dickinson, Franklin Lakes, USA).

**Microarray hybridization**

Biotinylated double strand cDNAs were prepared from the amplified total RNA. Following fragmentation and end-labelling, cDNAs were hybridized on GeneChip HG-U133 Plus 2.0 or Human Transcriptome Array 2.0 (Affymetrix, Santa Clara, CA, USA) for stromal cells or T cell subsets, respectively. For stromal cell data analysis, expression signal values were obtained for each probe by the Robust Multichip Averaging algorithm using GC content of probes with Partek software (Partek Incorporated, Saint Louis, MO, USA). The genes differentially between TSCs and FRCL were identified using a paired t-test carried out with Partek software (adjusted p-value<.05, log2 fold change>1). For T-cell subset data analysis, CEL files were further processed with Affymetrix Expression Console software version 1.4.1 using Robust Multi-array Average (RMA) algorithms with default settings at the gene and exon levels.

1. Amé-Thomas P, Maby-El Hajjami H, Monvoisin C, Jean R, Monnier D, Caulet-Maugendre S, Guillaudeux T, Lamy T, Fest T, Tarte K. Human mesenchymal stem cells isolated from bone marrow and lymphoid organs support tumor B-cell growth: role of stromal cells in follicular lymphoma pathogenesis. *Blood* (2007) **109**:693–702. doi:10.1182/blood-2006-05-020800
