# Supplementary material for: Human Lymphoid Stromal Cells Contribute to Polarization of Follicular T Cells Into IL-4 Secreting Cells
Source: Front Immunol. 2020 Oct 2;11:559866. doi: 10.3389/fimmu.2020.559866 (PMC7562812; doi:10.3389/fimmu.2020.559866)
Supplement: Supplementary file 6 [file Table_1.doc]

**Supplemental Table S1: List of antibodies**

| **Antigen** | **Supplier** | **Fluorochrome** | **Usage** | **Clone** |
| --- | --- | --- | --- | --- |
| CD3 | Becton Dickinson | PE-CF594 | FC | UCHT1 |
| CD4 | eBioscience | PerCP/Cy5.5 | FC | RPTA-4 |
| CD25 | Becton Dickinson | PE | FC | M-A251 |
| CD279 | eBioscience | PE/Cy7 | FC | eBioJ105 |
| CXCR5 | R&D Systems | APC | FC | 51505 |
| CD2 | Becton Dickinson | PE-CF594 | FC | RPA-2.10 |
| IL-4 | Becton Dickinson | PE | FC | 8D4-8 |
| IFN-y | eBioscience | eFluor 450 | FC | 4S.B3 |
| IL-21 | eBioscience | PE | FC | eBio3A3-N2 |
| CD54 | R&D Systems | - | B/N | BBIG-I1 |
| CD54 | Becton Dickinson | PE | FC | HA58 |
| CD106 | R&D Systems | - | B/N | BBIG-V1 |
| CD106 | eBioscience | PE | FC | STA |
| CD105 | Becton Dickinson | BV510 | FC | 266 |
| caspase 3 | Becton Dickinson | PE | FC | C92-605 |

B/N: blocking/neutralizing; FC: flow cytometry
