# Supplementary material for: Human Lymphoid Stromal Cells Contribute to Polarization of Follicular T Cells Into IL-4 Secreting Cells
Source: Front Immunol. 2020 Oct 2;11:559866. doi: 10.3389/fimmu.2020.559866 (PMC7562812; doi:10.3389/fimmu.2020.559866)
Supplement: Supplementary file 7 [file Table_2.doc]

**Supplemental Table S2. Assays on demand used for quantitative RT-PCR**

| **Gene symbol** | **Assay ID** |
| --- | --- |
| 18S | Hs99999901_s1 |
| AHR | Hs00169233_m1 |
| B2M | Hs99999907_m1 |
| BATF | Hs00232390_m1 |
| BCL6 | Hs00153368_m1 |
| CASC3 | Hs00904832_m1 |
| CXCL13 | Hs00757930_m1 |
| CXCR4 | Hs02330069_s1 |
| FOXP3 | Hs00203958_m1 |
| GPR183 | Hs00270639_s1 |
| ICOS | Hs00359999_m1 |
| IFNG | Hs00174143_m1 |
| IL10 | Hs00961622_m1 |
| IL17A | Hs00174383_m1 |
| IL17F | Hs00369400_m1 |
| IL21 | Hs00222327_m1 |
| IL21R | Hs00222310_m1 |
| IL22 | Hs01574154_m1 |
| IL2 | Hs00174114_m1 |
| IL4 | Hs00174122_m1 |
| ITGB1 | Hs00559595_m1 |
| ITGB2 | Hs00164957_m1 |
| LY9 | Hs03004330_m1 |
| MAF | Hs04185012_s1 |
| NOTCH1 | Hs01062014_m1 |
| NOTCH2 | Hs01050702_m1 |
| NRP1 | Hs00826128_m1 |
| POU2AF1 | Hs01573371_m1 |
| PRDM1 | Hs00153357_m1 |
| SELL | Hs00174151_m1 |
| SELPLG | Hs00380945_m1 |
| SH2D1A | Hs00158978_m1 |
| SLAMF1 | Hs00234149_m1 |
| SLAMF6 | Hs01559920_m1 |
| STAT3 | Hs00374280_m1 |
| STAT4 | Hs01028017_m1 |
| TBX21 | Hs00203436_m1 |
| TNF | Hs00174128_m1 |
| TNFRSF4 | Hs00937194_g1 |
| TNFSF13B | Hs00198106_m1 |
